# Editorial: Biological/chemical-based metallic nanoparticles synthesis, characterization, and environmental applications

**DOI:** 10.3389/fchem.2023.1191659

**Published:** 2023-04-04

**Authors:** Aziz Eftekhari, Rovshan Khalilov, Taras Kavetskyy, Cumali Keskin, Ram Prasad, Gvozden Luka Rosic

**Affiliations:** ^1^ Department of Biochemistry, Faculty of Science, Ege University, Izmir, Türkiye; ^2^ Department of Biophysics and Biochemistry, Baku State University, Baku, Azerbaijan; ^3^ Department of Biology and Chemistry, Drohobych Ivan Franko State Pedagogical University, Drohobych, Ukraine; ^4^ Department of Biology, Mardin Artuklu University Graduate Education Institute, Mardin, Türkiye; ^5^ Department of Botany, Mahatma Gandhi Central University, Bihar, India; ^6^ Department of Physiology, Faculty of Medical Sciences, University of Kragujevac, Kragujevac, Serbia

**Keywords:** bio nanoparticles, bioremediation, environmental applications, green synthesis, nanobiotechnology

Parts of plants are used to carry out the reduction reactions. Although there are different methods for the synthesis of nanomaterials, biological synthesis is relatively cheap, environmentally friendly, and safe compared to other methods (Ahmadov and Ramazanli, 2019; Ramazanli and Ahmadov, 2022). The aim of the Research Topic on “Biological/Chemical-Based Metallic Nanoparticles Synthesis, Characterization, and Environmental Applications” was to provide an integrated view of the state-of-the-artresearch on recent advances in biosynthesis, characterization of biological/chemical-based nanomaterials, and their application by providing a comprehensive understanding of the topic through original research and review articles focusing on the biological synthesis method, in which bacteria, fungi, algae, and various.

Here, we provide a brief editorial overview of the articles published on this Research Topic.

Following the cost-effective green chemistry technique that uses plants, Khalid and colleagues chose *Phyllanthus emblica* (gooseberry) leaf extract for the synthesis of boron-doped zinc oxide nanosheets as an antibacterial agent. Indeed, the applied methodology confirmed the efficacy of boron-doped zinc oxide nanosheets against Gram-negative bacteria (*K. pneumonia* and *E. coli*), and the linear attenuation coefficient was enhanced due to the increased amount of boron. They concluded that boron-doped zinc oxide nanosheets can be used as a radiation shielding material.

As a water-soluble xanthene dye, Rhodamine B (RhB) has also been considered an organic pollutant in the environment and has been extensively used as a trace dye for water flow in industry. Gami et al. confirmed that silver and gold nanoparticles based on *L. frutescens* (Berl.) I. M. Johnst (Scrophulariaceae) leaf extract catalytically degraded Rhodamine B dye in the presence of UV light. This is the first report on the preparation of green silver and gold nanoparticles with optimized temperature, extraction time, and metal precursor concentration using *Leucophyllum frutescens* leaf extract. They recommended this method for photodynamic and photothermal uses, and also for biomedical applications, including in cancer and antibacterial therapy.

Another study by Kumari Githala and colleagues showed that organic dyes such as Congo red and methylene blue were broken down using novel *Plantago ovata* leaf extract-based silver nanoparticles. They also found that these nanoparticles have potent antifungal activity against the growth of *Alternaria alternate*.

Another natural source of reducing and capping agents for the synthesis of nanoparticles is fungi, due to the presence of secondary metabolites and extracellular enzymes. The investigation by Gaba and colleagues showed the significant antifungal impact of copper oxide nanoparticles from *T. asperellum* culture filtrate on the growth of the tested pathogen, *A*. *brassicae.* Microscopic techniques showed that copper oxide nanoparticles induced vacuolization and disrupted the cytoplasmic functions in *A*. *brassicae*cells.

Arshad et al. synthesized silver nanoparticles based on *Periploca aphylla* Dcne. and coated these with poly (ethylene glycol) methacrylate for plant tissue culture in the biomass enhancement of *Stevia* rebaudiana calli. They observed a significant increase in biomass accumulation, and they suggested that poly (ethylene glycol) methacrylate-capped silver nanoparticles are suitable for the growth promotion and production of essential bioactive compounds from medicinal plants in the field of biotechnology.

Overall, the environmental applications of biological/chemical-based metallic nanoparticles have not been adequately assessed, and any work to determine how they function and develop potential defense mechanisms against environmental pollutants could greatly alleviate industrial problems.
